# Default mode network connectivity is associated with long-term clinical outcome in patients with schizophrenia

**DOI:** 10.1016/j.nicl.2019.101805

**Published:** 2019-04-01

**Authors:** Hyeongrae Lee, Dong-Kyun Lee, Kyeongwoo Park, Chul-Eung Kim, Seunghyong Ryu

**Affiliations:** a Department of Mental Health Research, National Center for Mental Health, Seoul, Republic of Korea; b Mental Health Research Institute, National Center for Mental Health, Seoul, Republic of Korea

**Keywords:** Schizophrenia, Outcome, Functional connectivity, Default mode network, Resting-state

## Abstract

This study investigated whether resting-state functional connectivity is associated with long-term clinical outcomes of patients with schizophrenia. Resting-state brain images were obtained from 79 outpatients with schizophrenia and 30 healthy controls (HC), using a 3 T-MRI scanner. All patients were 20–50 years old with >3 years' duration of illness and appeared clinically stable. We assessed their psychopathology using the 18-item Brief Psychiatric Rating Scale (BPRS-18) and divided them into “good,” “moderate,” and “poor” outcome (SZ-GO, SZ-MO, and SZ-PO) groups depending on BPRS-18 total score. We obtained individual functional connectivity maps between a seed region of the bilateral posterior cingulate cortex (PCC) and all other brain regions and compared the functional connectivity of the default mode network (DMN) among the HC and 3 schizophrenia outcome groups, with a voxel-wise threshold of P < .001 within a cluster-extent threshold of 114 voxels. Additionally, we assessed correlations between functional connectivity and BPRS-18 scores. The SZ-MO and SZ-PO groups showed decreased functional connectivity between PCC and right ventromedial prefrontal cortex (vmPFC), left middle cingulate cortex, and left frontopolar cortex (FPC) compared to the SZ-GO and HC groups. DMN connectivity in the right vmPFC and left FPC negatively correlated with subscale scores of the BPRS-18, except the negative symptoms subscale. In this study, poorer clinical outcomes in patients with schizophrenia were associated with decreased DMN connectivity. In particular, the decreased functional connectivity might be related to the severity of positive and mood symptoms rather than negative symptoms.

## Highlights

• Long-term outcome of schizophrenia is related to default mode network connectivity.
• Decreased functional connectivity is associated with poorer clinical outcome.
• Functional connectivity negatively correlates with positive and mood symptoms.

## 1 Introduction

Schizophrenia is a mental illness characterized by delusions, hallucinations, disordered formal thought, disorganized behaviors, negative symptoms, and cognitive dysfunctions (Kahn et al., 2015). The outcomes of schizophrenia vary widely among individuals, but the majority of patients with schizophrenia experience residual symptoms and impaired social functioning. The positive symptoms, such as delusions and hallucinations, tend to relapse and remit, although some patients suffer from treatment-resistant residual psychotic symptoms (Bertelsen et al., 2009). Even after patients achieve remission from psychotic symptoms, the negative symptoms and cognitive dysfunctions tend to be chronic, which leads to impairments in social and occupational functioning (Buchanan, 2007). In terms of course and outcome of schizophrenia, it is generally known that approximately one-third of patients have a relatively good outcome, with no more than mild symptoms and functional impairments, and the remaining two-thirds have moderate to severe symptoms and functional impairments (Menezes et al., 2006).

Neuroimaging markers associated with the clinical course and outcome of schizophrenia have been studied. Structural magnetic resonance imaging (sMRI) studies have shown that patients with poor outcomes have more marked reductions in total and regional gray matter volume, and greater ventricular enlargement, compared to those with good outcomes (Kubota et al., 2015; Wojtalik et al., 2017). Functional MRI (fMRI) studies have reported widespread network dysconnectivity in schizophrenia (Yu et al., 2012), but the relationship between dysconnectivity and long-term clinical course of schizophrenia remains unknown. Considering evidence of progressive loss of synaptic activity in patients with schizophrenia (Marsman et al., 2013), clinical progression or deterioration in schizophrenia might be associated with impaired control of synaptic plasticity leading to dysfunctional integration of neural systems, i.e., dysconnectivity (Friston, 1999; Friston and Frith, 1995; Stephan et al., 2006).

fMRI studies have demonstrated aberrant functioning of the default mode network (DMN) in schizophrenia. The DMN, a distributed network composed of a set of brain regions including the medial prefrontal cortex (mPFC), posterior cingulate cortex (PCC), precuneus, and lateral/medial temporal lobes (Fox and Raichle, 2007; Fransson, 2005), shows increased activation during rest or internal cognitive processing, but decreased activation during externally goal-directed cognitive tasks (Raichle et al., 2001). DMN alterations have also been associated with various symptoms of schizophrenia, including positive, negative, and disorganized symptoms (Camchong et al., 2011; Rotarska-Jagiela et al., 2010; Whitfield-Gabrieli et al., 2009). In addition, recent studies have reported reduced DMN suppression during a broad range of cognitive tasks in patients with schizophrenia, which could be interpreted as the cause or the consequence of cognitive deficits (Fornito et al., 2012; Pomarol-Clotet et al., 2008). Considering that persistent, unremitting psychotic symptoms, prominent negative symptoms, and more severe cognitive impairments are clinical factors that suggest poor prognosis (Lang et al., 2013), DMN abnormalities may be associated with the course and outcome of individuals with schizophrenia.

This study investigated the relationship between DMN connectivity and long-term clinical outcomes of schizophrenia. Accordingly, we performed PCC seed region connectivity analysis using resting-state functional MRI data and compared DMN connectivity among healthy controls (HCs) and 3 groups of patients with chronic schizophrenia who were considered to have good, moderate, and poor outcomes (SZ-GO, SZ-MO, and SZ-PO). In addition, we examined correlations between DMN connectivity and psychopathology in patients with schizophrenia.

## 2 Materials and methods

### 2.1 Subjects

All subjects were interviewed by a psychologist (K.P.), who used the Mini-International Neuropsychiatric Interview (MINI) (Sheehan et al., 1998) to make diagnoses of psychiatric disorders according to the Diagnostic and Statistical Manual of Mental Disorders, Fourth Edition (DSM-IV) (American Psychiatric Association, 1994). Patients who met the DSM-IV criteria for schizophrenia were recruited from outpatient clinics of the National Center for Mental Health, Seoul, Republic of Korea. Inclusion criteria were as follows: (1) aged 20–50 years; (2) duration of illness > 3 years; (3) appeared clinically stable, as defined by no exacerbation of psychotic symptoms and no change in general clinical state and medication for at least 3 months prior to the time of assessment. We excluded patients with a concurrent axis I diagnosis according to the DSM-IV, current or past neurological disease, any contraindication to MRI scan, or a physical condition that would render an MRI scan difficult to administer or interpret, e.g., severe extrapyramidal symptoms. The HCs consisted of volunteers from the local community who were free of any history of psychiatric disorders. The same exclusion criteria that were applied to the patients also applied to the HC group with regard to medical, neurological, and physical conditions. Data from 79 patients and 30 HCs were included in the final analysis.

This study was initiated after approval of the Institutional Review Board of the National Center for Mental Health (IRB approval number: 116271–2017-26), and written informed consent was obtained from all subjects.

### 2.2 Clinical assessments and group assignment

We defined long-term outcome of schizophrenia as overall severity of residual psychopathology in clinically stable patients with chronic schizophrenia whose clinical progression was considered to have reached a plateau after 3 years of onset. The overall level of the patient's psychopathology was evaluated by a psychiatrist (S.R.), who used the 18-item Brief Psychiatric Rating Scale (BPRS-18) (Overall and Gorham, 1962). Leucht et al. (2005) proposed that “mildly ill” according to the Clinical Global Impression (CGI) scale corresponds to BPRS-18 total scores of 18–31 and “moderately ill” corresponds to BPRS-18 total scores of 32–41. Based on these criteria, we classified the patients into 3 groups: SZ-GO (BPRS-18 total scores of 18–31, N = 25), SZ-MO (BPRS-18 total scores of 32–41, N = 31), and SZ-PO (BPRS-18 total scores of ≥42, N = 23). In addition, according to the factor structure proposed by Shafer (2005), the “affect,” “positive symptoms,” “negative symptoms,” “resistance,” and “activation” subscales of the BPRS-18 were analyzed as continuous variables. We also assessed the overall level of the patient's functioning using the WHO Disability Assessment Schedule 2.0 (WHODAS 2.0) (Gold, 2014). Considering that functional outcomes of patients with schizophrenia correlate with their clinical outcomes, we compared the WHODAS 2.0 total score among SZ-GO, SZ-MO, and SZ-PO groups to confirm whether the grouping was appropriate.

### 2.3 MRI acquisition

The MRI data were acquired using a 3-T MR scanner (Ingenia CX; Philips, Erlangen) equipped with a 32-channel head coil. Blood oxygen-level dependent (BOLD) signals were obtained using a T2*-weighted gradient echo planar imaging (EPI) sequence (TR = 2000 ms, TE = 30 ms, matrix size: 64 × 64, 45 interleaved slices, field of view = 220 mm, slice thickness = 3.5 mm, flip angle = 90°) parallel to the anterior commissure-posterior commissure line. All subjects received a 5-min scan (150 volumes) during functional imaging plus two initial dummy volumes, which were acquired to ensure magnetization stabilization. During the functional scans, the participants were explicitly instructed to open their eyes and fixate on a white cross presented in the center of visual field. A high-resolution anatomical T1 weighted image was also acquired using a turbo field echo sequence (spin-echo TR = 9.8 ms, TE = 4.6 ms, matrix size: 240 × 240, 170 sagittal slices, FOV = 240 mm, slice thickness = 1 mm, flip angle = 8°).

### 2.4 Resting-state fMRI analysis

The fMRI data analysis was performed using the Analysis of Functional NeuroImages (AFNI) software (version 18.2.04) (Cox, 1996). The first three volumes were discarded from the functional dataset to allow for signal stabilization. Despiking was performed for each voxel time-series to suppress local spikes in the EPI signals. Head motion correction was conducted by rigid body registration of EPI volumes to a base EPI volume. Physiological respiratory and cardiac artifacts were estimated and corrected using Physiologic EStimation by Temporal ICA (PESTICA) (Beall and Lowe, 2007). Slice-timing correction was carried out for all slices within a volume. After co-registration to high-resolution anatomical T1 images, spatial normalization was performed by affine transform, using the parameters obtained from spatial normalization of the T1 images into Montreal Neurological Institute (MNI) stereotactic space with the MNI avg152T1 template. All voxels were resampled to 2 × 2 × 2 mm by nearest neighbor interpolation. A temporal band-pass filter was applied at 0.009 Hz < *f* < 0.08 Hz. The nuisance signals, which consisted of six parameters obtained by correction of head motion and non-neural sources of variance from the eroded white matter and the eroded large ventricle mask, were regressed out. Additionally, outlier volumes were censored from the time-series based on a threshold of framewise displacement, which was taken as the sum of the absolute value of temporal differences across the six motion parameters (Power et al., 2014). The time-points with a framewise displacement that exceeded 0.5 mm, also including the one preceding and following time-points, were excluded. Spatial smoothing was conducted with an isotropic Gaussian kernel of 7 mm full width at half-maximum.

In order to identify the default mode network, we performed seed-based functional connectivity analysis using the bilateral PCC (MNI coordinate x = ±6, y = 54, z = 26, radius = 6 mm). Individual functional connectivity maps were produced by correlation analysis between the average time course in the seed region and the time courses from the whole brain. The correlation coefficients were converted into z-values as functional connectivity strengths, using Fisher's r-to-z transformation.

### 2.5 Statistical analyses

To identify within-group functional connectivity patterns, a one-sample *t*-test was performed. Then, we explored brain regions showing differences in DMN connectivity among the HC and 3 schizophrenia outcome groups using analysis of covariance (ANCOVA), adjusting for sex, age, and education in the AFNI software. Significance thresholding for group analysis was determined using 3dClustSim, as available in the AFNI software suite. We adopted a voxel-wise threshold of P < .001 within a cluster extent threshold of 114 voxels, which corresponded to a family-wise error corrected P < .01.

In the regions showing significant differences among the 4 groups, we extracted mean values of functional connectivity strength. Then, we examined differences in DMN connectivity between pairs of groups using Bonferroni post-hoc test after ANCOVA adjusting for sex, age, and education, using the Statistical Package for the Social Science (SPSS) version 21.0 (IBM Corp., Armonk, NY, USA). In addition, we performed partial correlation analyses between mean values of DMN connectivity and BPRS-18 subscale scores, adjusting for sex, age, education, and chlorpromazine equivalent dose of antipsychotic drug (only in patients with schizophrenia). The partial correlation analyses were also performed using SPSS. For the post-hoc tests and partial correlation analyses, P < .05 was considered statistically significant.

## 3 Results

### 3.1 Demographic and clinical characteristics

Of the 79 patients with schizophrenia, 43 (54.43%) were men, and the mean age at study enrollment was 38.65 ± 7.31 years. The mean duration of illness was 16.52 ± 7.67 years. A high proportion of the patients (N = 51, 64.56%) had received antipsychotic polypharmacy. Risperidone was the most frequently prescribed antipsychotic drug (N = 25), followed by quetiapine (N = 20), olanzapine (N = 19), aripiprazole (N = 19), clozapine (N = 18), paliperidone (N = 12), blonanserin (N = 7), amisulpride (N = 6), ziprasidone (N = 5), sulpiride (N = 4), zotepine (N = 3), haloperidol (N = 2), chlorpromazine (N = 2), and perphenazine (N = 1). On average, each patient had been treated with 837.61 mg chlorpromazine equivalent dose of antipsychotic drug.

Table 1 summarizes the demographic and clinical characteristics of the HC and 3 schizophrenia outcome groups. There was no difference in age and sex among the 4 groups, but HCs had a significantly higher level of education than the SZ-MO (post-hoc P = .015) and SZ-PO (post-hoc P = .023) groups. Treatment dose of antipsychotic drug was significantly higher in the SZ-PO group than in the SZ-GO group (post-hoc P = .015). The WHODAS 2.0 score was lowest in the SZ-GO group, followed by the SZ-MO group, and highest in the SZ-PO group (F = 35.84, P < .001).

**Table 1:** Demographic and clinical characteristics of study participants.

Variables^a^	Healthy controls (N = 30)	Outcome groups of patients with schizophrenia			Statistics^b^
		Good (N = 25)	Moderate (N = 31)	Poor (N = 23)	
Age, y	37.60 ± 7.02	41.00 ± 7.07	37.77 ± 7.51	37.26 ± 6.97	F = 1.50, P = .220
Sex (male / female), n	15 (50.0) / 15 (50.0)	15 (60.0) / 10 (40.0)	16 (51.6) / 15 (48.4)	12 (52.2) / 11 (47.8)	χ^2^ = 0.63, P = .890
Education, y	14.77 ± 3.15	13.00 ± 3.25	12.68 ± 1.66	12.61 ± 2.17	F = 4.32, P = .007
Duration of illness, y	–	16.56 ± 7.01	16.42 ± 8.17	16.61 ± 8.00	F < 0.01, P = 1.00
Antipsychotic dose (chlorpromazine equivalent), mg	–	626.81 ± 394.17	860.83 ± 464.93	1035.43 ± 605.26	F = 4.22, P = .018
BPRS-18 total score	–	26.60 ± 2.92	36.52 ± 2.80	46.70 ± 3.47	F = 260.98, P < .001
Affect subscale	–	5.48 ± 1.19	7.58 ± 2.54	8.78 ± 2.88	F = 12.59
					P < .001
Positive symptoms subscale	–	6.00 ± 1.87	9.71 ± 2.34	12.83 ± 2.08	F = 62.12
					P < .001
Negative symptoms subscale	–	7.24 ± 2.13	9.00 ± 2.31	12.09 ± 2.13	F = 29.65
					P < .001
Resistance subscale	–	3.32 ± 0.56	5.00 ± 1.53	6.83 ± 2.57	F = 25.12
					P < .001
Activation subscale	–	3.56 ± 1.04	4.23 ± 1.02	5.17 ± 1.07	F = 14.41
					P < .001
WHODAS 2.0 total score	–	13.17 ± 5.29	17.87 ± 6.51	28.14 ± 6.82	F = 35.84, P < .001

Abbreviations: BPRS-18, 18-item Brief Psychiatric Rating Scale; WHODAS 2.0, WHO Disability Assessment Schedule 2.0.

a Mean ± SD or N(%).

b ANOVA or chi-square test.

### 3.2 Comparison of functional connectivity among HC and 3 clinical outcome groups

**Fig. 1:**
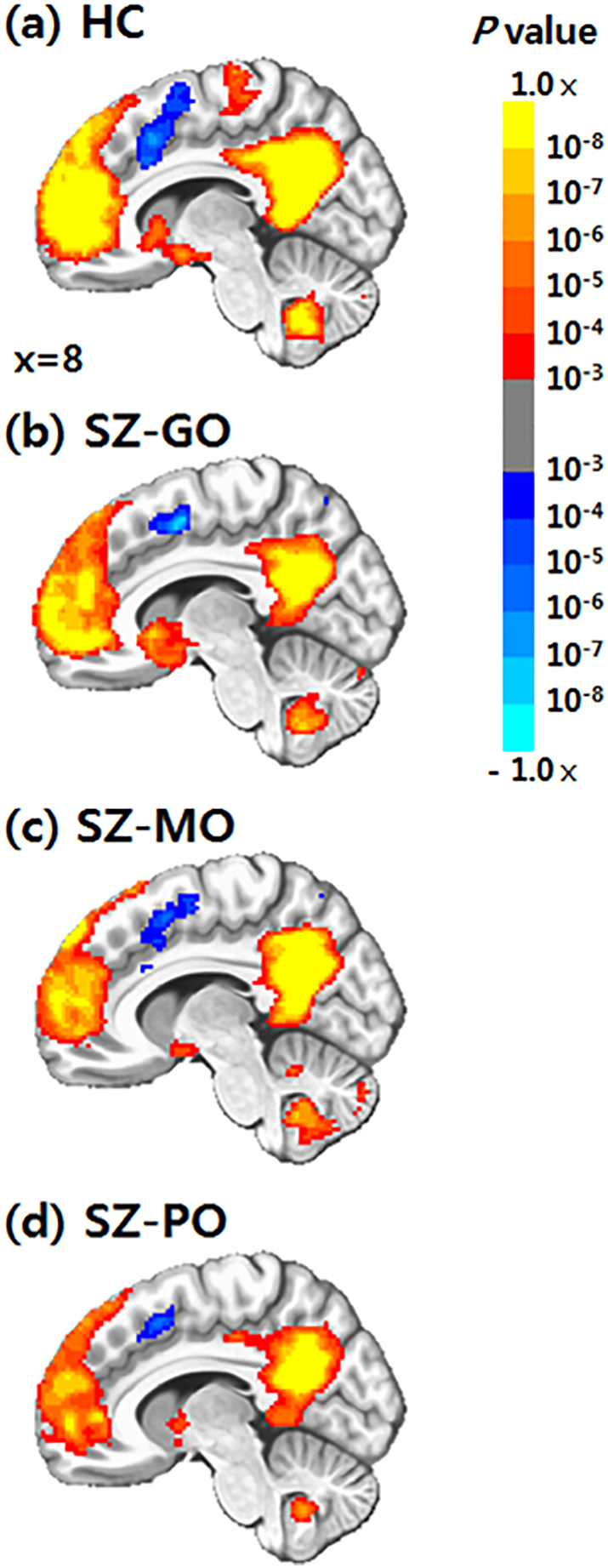
Brain areas showing functional connectivity with the seed region of the bilateral posterior cingulate cortex. Abbreviations: HC, healthy controls; SZ-GO, schizophrenia patients with good outcome; SZ-MO, schizophrenia patients with moderate outcome; SZ-PO, schizophrenia patients with poor outcome.

**Fig. 2:**
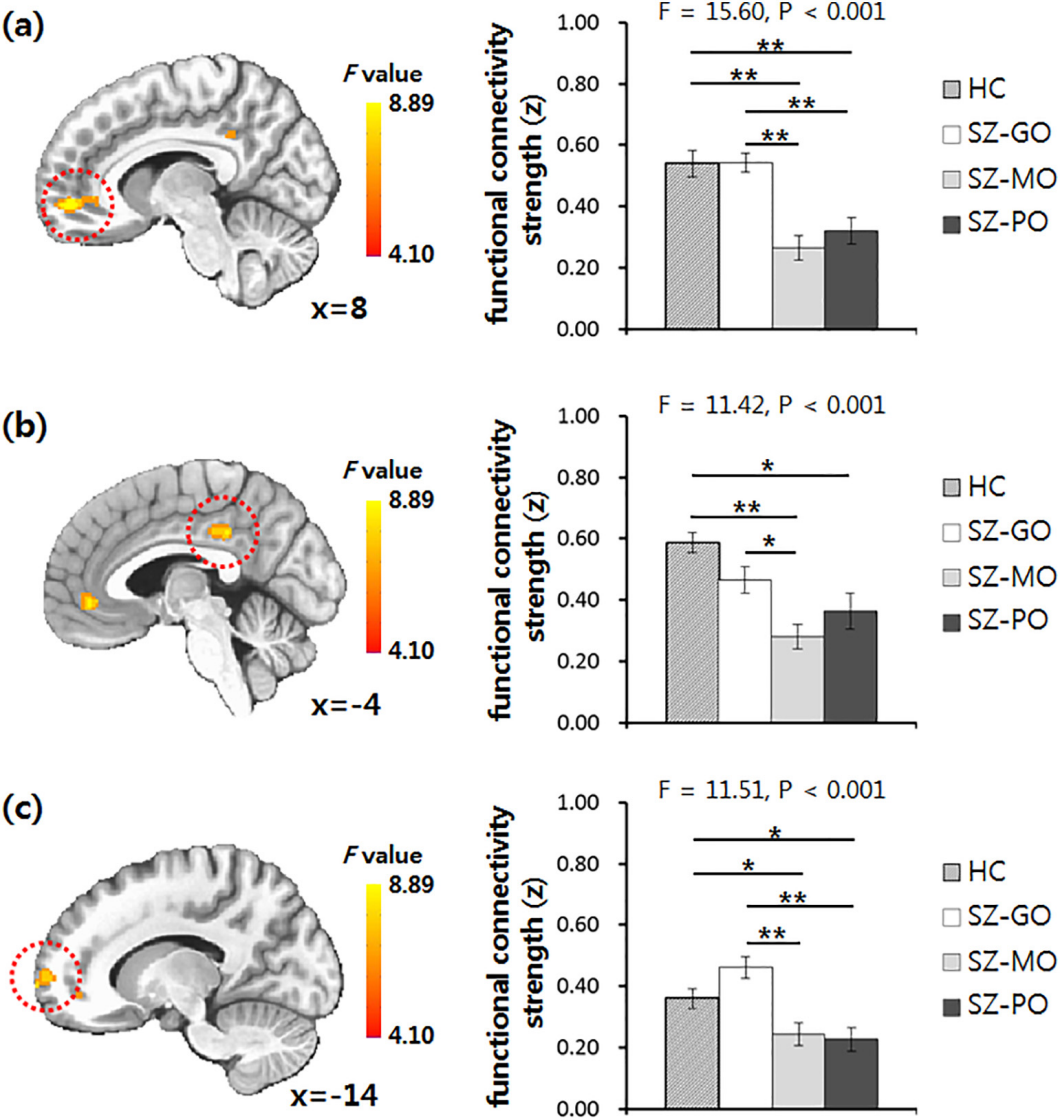
Group comparisons of default mode network connectivity using analysis of covariance and Bonferroni post-hoc test. Red circles indicate (a) right ventromedial prefrontal cortex, (b) left middle cingulate cortex, and (c) left frontopolar cortex, the regions showing significant differences in functional connectivity with the seed region of the bilateral posterior cingulate cortex. The group difference brain maps are superimposed onto the MNI152 T1 template. Abbreviations: HC, healthy controls; SZ-GO, schizophrenia patients with good outcome; SZ-MO, schizophrenia patients with moderate outcome; SZ-PO, schizophrenia patients with poor outcome. *Post-hoc P < .05, **post-hoc P < .001.

Fig. 1 depicts DMN connectivity in each group of HCs and patients with schizophrenia. We observed significant differences in functional connectivity between the PCC and right ventromedial prefrontal cortex (vmPFC), left middle cingulate cortex (MCC), and left frontopolar cortex (FPC) among the HC and 3 schizophrenia outcome groups (Table 2). In these regions, ANCOVA also revealed significant differences in mean values of functional connectivity strength among the 4 groups (right vmPFC: F = 15.60, P < .001; left MCC: F = 11.42, P < .001; left FPC: F = 11.51, P < .001). Post-hoc tests confirmed that the functional connectivity between PCC and right vmPFC significantly decreased in the SZ-MO and SZ-PO groups compared to the HC (post-hoc P < .001, all) and SZ-GO groups (post-hoc P < .001, all) (Fig. 2). DMN connectivity in the left MCC was significantly lower in the SZ-MO (post-hoc P < .001) and SZ-PO (post-hoc P = .001) groups than in the HC group. Compared to the SZ-GO group, the SZ-MO group showed significantly decreased DMN connectivity in the left MCC (post-hoc P = .003). DMN connectivity in the left FPC significantly decreased in the SZ-MO and SZ-PO groups compared to the HC (post-hoc P = .004 and 0.003, respectively) and SZ-GO (post-hoc P < .001, all) groups. In addition, we observed no difference in DMN connectivity between HC and SZ-GO groups or between SZ-MO and SZ-PO groups.

**Table 2:** Regional differences in default mode network connectivity among healthy controls and 3 outcome groups of patients with schizophrenia.

Anatomical region	Side	Peak coordinates (MNI)			Cluster size (voxels)	F max
		x	y	z		
Ventromedial prefrontal cortex	R	8	54	−6	311	8.893
Middle cingulate cortex	L	−4	−36	36	133	6.444
Frontopolar cortex	L	−14	66	4	123	6.107

Abbreviations: MNI, Montreal Neurological Institute; R, right; L, left.

### 3.3 Relationships between functional connectivity and psychopathology

With respect to the 5 subscales of the BPRS-18, partial correlation analysis revealed that DMN connectivity in the right vmPFC negatively correlated with affect (*r* = −0.30, P = .010), positive symptoms (*r* = −0.26, P = .025), resistance (*r* = −0.29, P = .013), and activation (*r* = −0.32, P = .005) subscale scores (Table 3). DMN connectivity in the left FPC also negatively correlated with positive symptoms (*r* = −0.33, P = .004) and resistance (*r* = −0.39, P = .001) subscale scores.

**Table 3:** Correlations between default mode network connectivity and psychopathology of schizophrenia^a^.

Anatomical region	Subscale scores of BPRS-18				
	Affect	Positive	Negative	Resistance	Activation
Ventromedial prefrontal cortex					
*r*	−0.30	−0.26	−0.21	−0.29	−0.32
P value	0.010	0.025	0.078	0.013	0.005

Middle cingulate cortex					
*r*	−0.11	−0.16	0.02	−0.10	−0.05
P value	0.351	0.173	0.840	0.401	0.648

Frontopolar cortex					
*r*	−0.22	−0.33	−0.22	−0.39	−0.20
P value	0.055	0.004	0.055	0.001	0.087

Abbreviations: BPRS-18, 18-item Brief Psychiatric Rating Scale; *r*, partial correlation coefficient.

a Partial correlation analysis, adjusting for sex, age, education, and dose of antipsychotic drug.

## 4 Discussion

Resting-state functional connectivity of the DMN in schizophrenia has been extensively studied. However, few investigations have reported correlations between DMN connectivity and clinical or psychosocial outcomes in schizophrenia. To the best of our knowledge, this study is the first to investigate the associations between DMN connectivity and long-term clinical outcomes of schizophrenia. In this study, the DMN maps of patients with poorer outcome differed significantly from those of not only patients with better outcome but also healthy people. The differences in connectivity patterns of the DMN were observed in the right vmPFC, left MCC, and left FPC, which have been associated with the pathophysiology of schizophrenia. In addition, the present study found that the degree of alteration in functional connectivity in the prefrontal cortices correlated with the severity of positive and mood symptoms in patients with schizophrenia.

In this study, patients with poorer clinical outcome demonstrated reduced DMN connectivity in the medial frontal areas, compared to healthy subjects. However, there was no difference in DMN connectivity between patients with better outcome and healthy subjects. Altered resting-state functional connectivity of the DMN in schizophrenia has been reported, but the results have been mixed. Bluhm et al. (2007) examined functional connectivity of the DMN using a seed-based approach and reported reduced PCC connectivity with the lateral parietal region, mPFC, precuneus, and cerebellum in schizophrenia. Some studies using independent component analysis have revealed reduced DMN connectivity in medial frontal areas (Camchong et al., 2011; Rotarska-Jagiela et al., 2010). Our findings are partly consistent with these studies that reported hypo-connectivity of the DMN in schizophrenia. However, in contrast to these studies, several studies have observed hyper-connectivity of the DMN in schizophrenia. Whitfield-Gabrieli et al. (2009) found increased DMN connectivity between the PCC and mPFC during resting blocks of a working memory task. Woodward et al. (2011) reported increased PCC connectivity with the left inferior frontal gyrus, left middle frontal gyrus, and left middle temporal gyrus. Subsequent studies have replicated the findings of hyper-connectivity of the DMN in schizophrenia (Mannell et al., 2010; Salvador et al., 2010; Skudlarski et al., 2010). This inconsistency in DMN abnormalities across studies may be due to the marked differences in analytic techniques, small sample sizes, and heterogeneity of subject groups.

Considering that a considerable proportion of patients with schizophrenia do not reach remission of psychotic symptoms (Bertelsen et al., 2009), we postulated that unremitting clinical course is an important indicator of long-term outcome in schizophrenia. For the cross-sectional assessment, we defined long-term outcome of schizophrenia as overall severity of residual symptoms in clinically stable patients with chronic schizophrenia. We also observed that the groups with worse clinical outcome showed more functional disability, implying that our classification of long-term outcome based on psychopathologic severity was appropriate. In the current study, patients with poorer clinical outcome demonstrated reduced DMN connectivity in the medial frontal areas compared to those with better outcome. Although there is no comparative study of long-term clinical outcome in schizophrenia, associations between DMN connectivity and social functioning have been reported by Fox et al. (2017), who observed that stronger connectivity between PCC and mPFC was associated with better social attainment and social competence in patients with chronic schizophrenia; these results are consistent with our findings. Thus, our results suggest that alterations of DMN connectivity in the medial frontal areas might reflect outcome status in patients with schizophrenia. In addition, the groups with poorer outcome might have included many patients with treatment-resistant schizophrenia, who had experienced a considerable level of psychotic symptoms despite longstanding antipsychotic treatment. Therefore, the DMN abnormalities in these regions might be related to treatment-resistance in schizophrenia. Indeed, widespread reduction in resting state functional connectivity has been associated with treatment-resistant schizophrenia (Ganella et al., 2017).

DMN alterations have been associated with various types of symptoms in schizophrenia. Camchong et al. (2011) reported that frontal connectivity was positively associated with positive and negative symptoms. Rotarska-Jagiela et al. (2010) found negative correlations between hippocampus connectivity in the DMN and severity of hallucinations and delusions. Hyper-connectivity of the DMN has also been associated with worse symptoms of schizophrenia. Whitfield-Gabrieli et al. (2009) found that task suppression in mPFC negatively correlated with positive and negative symptoms. Woodward et al. (2011) observed a significant positive correlation between connectivity between PCC and left middle frontal gyrus and overall psychopathology. Our findings are consistent with previous studies that reported associations with hypo-connectivity of the DMN. We found that DMN connectivity in the prefrontal cortices negatively correlated with the severity of positive and mood symptoms in patients with schizophrenia, but did not observe any significant correlation with the severity of negative symptoms. Considering that the group with poorer clinical outcome showed higher severity of all subscale scores (Table 1), the association between hypo-connectivity of the DMN and poorer long-term outcome might primarily result from positive and mood symptoms, not negative symptoms.

In summary, this study suggests that aberrant functional connectivity between prefrontal cortices and PCC might be a neural underpinning of unremitting clinical course in schizophrenia. This is broadly consistent with findings of Garrity et al. (2007), which indicated altered modulation of the anterior and posterior cingulate cortices in schizophrenia. However, our seed-based analysis found only reduced temporal correlations between the DMN regions in schizophrenia; that is, we could not make inferences about directed and weighted connections among neural sources. In this regard, effective connectivity analysis may be helpful in understanding the cortical hierarchies and distributed processes. Bastos-Leite et al. (2015) used stochastic dynamic causal modelling (sDCM) of fMRI data to show that the excitatory influence of the posterior cingulate node on the anterior frontal node and the intrinsic inhibitory self-connection of the anterior cingulate node in the DMN were reduced in patients with schizophrenia. The reduced extrinsic connectivity in the sDCM study is consistent with hypo-connectivity of the DMN in this study. Moreover, as reduced self-inhibition in the anterior frontal node of the DMN could lead to increased excitability, the sDCM study's findings are also relevant for interpretation of hyper-connectivity of the DMN reported in several previous studies (Whitfield-Gabrieli et al., 2009; Woodward et al., 2011).

This study is subject to some methodological limitations. First, because we evaluated long-term outcome of patients with schizophrenia cross-sectionally, we could not determine whether the DMN abnormalities were state-markers or trait-markers of the outcome of schizophrenia. Second, there were differences in the level of education and daily dose of antipsychotic drug between groups. Although we statistically controlled for these covariates, we could not exclude the possibility that these differences influenced the results. Third, the sample size for each group was relatively modest, although reference maps derived from a large dataset were used. Taking these limitations into account, careful attention should be paid to interpretation of our findings.

In conclusion, we found an association between functional connectivity of the DMN and unremitting clinical outcome in schizophrenia. Our findings suggest that aberrant DMN connectivity may be a predictive biomarker of long-term clinical outcome of schizophrenia. Future longitudinal imaging studies to confirm that these alterations of the DMN predict long-term outcome of schizophrenia are warranted.
